# “It has changed my life”: unconditional cash transfers and personalized infant feeding support- a feasibility intervention trial among women living with HIV in western Kenya

**DOI:** 10.1186/s13006-023-00600-1

**Published:** 2023-11-27

**Authors:** Emily L. Tuthill, Ann E. Maltby, Belinda C. Odhiambo, Thomas J. Hoffmann, Maureen Nyaura, Rosemary Shikari, Craig R. Cohen, Sheri D. Weiser

**Affiliations:** 1 Department of Community Health Systems, School of Nursing, University of California San Francisco, San Francisco, CA USA; 2 Global Programs for Research and Training, University of California San Francisco, Kisumu, Kenya; 3 Department of Epidemiology and Biostatistics, and Office of Research School of Nursing, University of California San Francisco, San Francisco, CA USA; 4 Ambercare Medical Centre and Mamatoto Childbirth and Breastfeeding Educative Services, Kisumu, Kenya; 5 Department of Obstetrics, Gynecology & Reproductive Sciences, University of California San Francisco, San Francisco, CA USA; 6 Division of HIV, Infectious Disease, and Global Medicine, Department of Medicine, University of California San Francisco, San Francisco, CA USA

**Keywords:** Exclusive breastfeeding, Financial insecurity, Food insecurity, Unconditional cash transfers, HIV, Kenya

## Abstract

**Background:**

The syndemic effects of poverty, food insecurity and living with HIV are recognized as global health priorities, including through the United Nations Sustainability Goals 1, 2 and 3. Today, women and girls account for 63% of all new HIV infections in eastern and southern Africa, including Kenya. Pregnant and postpartum women living with HIV in this setting face unique challenges including increased financial insecurity as women leave the work force to care for their newborn infants. This contributes to poverty, food scarcity and stress.

**Methods:**

To address financial insecurity, improve infant feeding and reduce stress among mothers living with HIV in this setting, we developed a multilevel intervention, Supporting Healthy Mothers, consisting of 10 monthly unconditional cash transfers (10,000 KES, ~$75 USD/month) and personalized infant feeding support from pregnancy to 7 months postpartum. We conducted a non-randomized feasibility trial of this intervention among women engaged in HIV care in Kisumu, Kenya. From February 23, 2022 to March 23, 2022, we enrolled a total of 40 women who were 20–35 weeks pregnant—20 women to the intervention group at a public clinic, and 20 women to the control group at a similar clinic. Our aim was to assess feasibility, acceptability, and the potential impact of the intervention on food security, infant feeding and maternal mental health.

**Results:**

Analyzing data from all 40 participants, we found a significant reduction in food insecurity scores from baseline for the intervention group when compared to the control group at 6 weeks and 6 months postpartum (*p* = 0.0008 and *p* < 0.0001, respectively). Qualitative exit interviews with intervention group participants confirmed women felt more financially secure and had newly acquired practical knowledge and skills related to infant feeding. Women found the two intervention components highly acceptable and described an overall positive impact on wellbeing.

**Conclusions:**

The Supporting Healthy Mothers intervention has potential to positively impact women across the perinatal period and beyond by increasing financial security and supporting women to overcome infant feeding challenges and should be assessed in larger trials.

**Trial registration:**

Supporting Healthy Mothers was registered with ClinicalTrials.gov Protocol Registration and Results System, initially published on February 1, 2022. ClinicalTrials.gov ID: NCT05219552 Protocol ID: K23MH116807.

## Background

The United Nations Sustainable Development Goals 1, 2 and 3 prioritize ending poverty and hunger and promoting good health and wellbeing for all [1]. Despite great progress, achieving these goals remains an urgent matter for sub-Saharan Africa given a disproportionate number of people suffer from extreme poverty and severe food insecurity in this region, which simultaneously bears an unequal share of the global HIV disease burden [2, 3]. Women and girls now account for 63% of all new HIV infections in sub-Saharan Africa [4]. In addition, lower education levels and less access to paid work and property ownership make women more likely to experience poverty [2]. The combined challenges of poverty, food insecurity and living with HIV are especially difficult for pregnant and postpartum (perinatal) women, many of whom are newly infected during pregnancy or while breastfeeding [4].

During the perinatal period, women have increased dietary needs and are generally less able to perform paid work as they face physical limitations and must dedicate time to caring for their infant [5, 6]. Perinatal women living with HIV (WLWH) also worry about the possibility of transmitting HIV to their baby which can occur during pregnancy, childbirth or during breastfeeding [7]. Optimal nutrition, including breastfeeding, is of critical importance to the health of HIV exposed infants, and as such, it is a chief concern for mothers in this setting [8, 9]. Current evidence-based guidelines universally recommend exclusive breastfeeding for the first 6 months of life followed by continued breastfeeding along with safe and adequate complementary feeding until 24 months or beyond [7, 10]. Yet, in sub-Saharan Africa, including Kenya, adherence to these guidelines is threatened by many factors such as contradictory cultural norms, worries about transmitting HIV through breastmilk and limited access to food and water [11–16].

In Kisumu County, Kenya, the prevalence of HIV among women (15–49 years old) is approximately 17.4% (compared to 5.2% among women nationally), and the rate of mother to child transmission of HIV is 8.7% with 87% of perinatal WLWH engaged in care at prevention of mother to child transmission of HIV (PMTCT) clinics [17]. Longitudinal exploratory research, among WLWH engaged in HIV care in Kisumu, has revealed that financial and food insecurity negatively impact women’s mental health and wellbeing and act as central barriers to optimal infant feeding for HIV exposed infants across time [18].

To address financial and food insecurity and sub-optimal infant feeding for HIV exposed infants while supporting the health and well-being of WLWH, we applied Transitions Theory [19] to develop a multilevel intervention incorporating unconditional cash transfers and personalized infant feeding support—Supporting Healthy Mothers. We conducted a feasibility trial of Supporting Healthy Mothers to assess the feasibility of implementing the intervention, the acceptability of the intervention’s components and trends in our primary outcomes, including food insecurity, mental health and breastfeeding behaviors.

## Methods

### Design and setting

We applied mixed-methods in this non-randomized feasibility trial, which took place at two PMTCT clinics within public hospitals in Kisumu. The clinics were similar in terms of size, services offered and socioeconomic status of the population served. Twenty participants from one clinic were enrolled into the intervention group and 20 to the control group at a second clinic (*N* = 40). Separate clinics were used for each group to ensure there was no social interaction between control and intervention group participants.

### Sample size

Sample size was determined based on standardized feasibility procedures [20, 21].

### Participants

Recruitment was carried out between February 23, 2022 and March 23, 2022. Women greater than 18-years-old, 20–35 weeks pregnant (based on last menstrual period), living with HIV and currently prescribed antiretroviral therapy (ART) were included. Women were excluded if they were having a high-risk pregnancy for reasons other than HIV status (e.g., pregnancy complications, preeclampsia, gestational diabetes, preterm labor), already participating in another ART adherence-related study, unable to understand the consent process, or planning to relocate out of the greater Kisumu area within 12 months.

Clinic staff conducted an initial screening of all pregnant WLWH being seen at the clinic. Interested women were then referred to our on-site research coordinators who met individually in a private room with women to verify their eligibility and interest to participate in the trial; women who remained willing and able to participate provided written informed consent.

### Supporting Healthy Mothers intervention

The Supporting Healthy Mothers intervention consisted of two components: 1) unconditional cash transfers, and 2) personalized infant feeding support. To develop the intervention we utilized previous research and applied Transitions Theory as a theoretical framework [19, 22]. The unconditional cash transfer was delivered to women via M-PESA, a mobile phone-based money transfer system that links money to a telephone number. Each intervention group participant received 10 monthly payments of 10,000 Kenyan Shillings (KES; approx. $75USD) beginning 1 month after enrollment until around 7 months postpartum. The number of monthly payments and targeted period of time were based on previous research which identified late pregnancy and early postpartum as the time of greatest financial insecurity for perinatal WLWH [23]. The amount to be given, frequency, and the form of payment were based the estimated range of income from informal employment in Kisumu, previously collected data about women’s estimated expenses, and previous studies involving cash transfers in this setting [24–26].

The second intervention component consisted of five personalized, in-person, infant feeding support sessions. The sessions were delivered by a locally trained, experienced doula and lactation specialist who assessed women’s practices, addressed challenges, and provided teaching about optimal infant feeding and care. The first infant feeding support session was delivered during pregnancy shortly after enrollment, and follow-up sessions occurred at 2, 4, 6 and 12 weeks postpartum. Detailed information about the lactation sessions is outlined in a separate publication [27].

Intervention group participants completed surveys using REDCap at enrollment, 2 weeks, 4 weeks, 6 weeks, 12 weeks, and 6 months postpartum. In addition, at each postpartum encounter, infant weights were measured [28–31]. To better understand women’s experiences, and gain feedback on the acceptability and perceived impact of the intervention, we conducted qualitative, semi-structured exit interviews at 7 months postpartum. This timing was chosen in order to capture women’s experiences with complementary feeding—a component of the infant feeding support sessions which most women initiated after 6 months of breastfeeding exclusively.

At each of the eight research encounters, participants received refreshments and were reimbursed 800KES ($5.80 USD) for their transportation expenses (in addition to the cash transfer).

### Control group

Women who participated in the control group received standard perinatal and PMTCT care. Upon enrollment and at 2 weeks, 4 weeks, 6 weeks, 12 weeks and 6 months postpartum, women completed electronic surveys, and at each postpartum encounter, infant weights were measured [28–31]. Women in the control group were provided refreshments and transportation reimbursement (800KES/$5.80 USD) at the six research encounters.

### Outcomes

#### Food insecurity

The Household Food Insecurity Access Scale (HFIAS), which has been successfully applied in similar Eastern African settings [32, 33], was used to measure food insecurity [34]. We looked at HFIAS scores as both a continuous variable (range 0 to 27 with higher scores indicating greater food insecurity) and when scores were categorized as food secure, mildly food insecure, moderately food insecure, or severely food insecure.

#### Mental health

We used the Patient Health Questionnaire-9 (PHQ-9) to assess for symptoms of depression, including anhedonia, depressed mood, insomnia, fatigue, appetite disturbance, guilt, diminished ability to think, psychomotor disruption, and suicidal ideation. The PHQ-9 uses a 4-point Likert Scale with scores ranging from 0 to 27 with higher scores corresponding to more depressive symptoms [35]. Women’s perceived stress was measured using the Perceived Stress scale-10 (PSS-10) consisting of 10 items which are responded to using a 5-point Likert scale. Score can range from 0 to 40 with higher scores indicating greater perceived stress [36, 37].

#### Breastfeeding behaviors

Women self-reported their breastfeeding behaviors via electronic surveys. We posed a variety of direct questions at various timepoints and allowed for explanations to be recorded along with the multiple choice/ select all that apply survey responses.

### Statistical analysis

#### Descriptive statistics

Frequencies, or means and standard deviations (or medians and interquartile ranges (IQR), if skewed) were calculated to describe food insecurity, depressive symptoms and perceived stress at three timepoints.

#### Association of group status with outcomes

To test for an association of these scores with the intervention versus control condition we utilized linear mixed models (LMM) with the package lme4 v1.1.27.1 [38] in R v4.1.0 [39] utilizing a non-parametric bootstrap (10,000 iterations; percentile confidence intervals) with the package boot v1.3.28 [40, 41] to handle slight deviations from normality. Covariates included whether intervention (vs. control), the time-point measured, and the interaction term between them (main coefficient of interest, measures if intervention differs from controls, from baseline).

### Exit interview analysis

**Fig. 1:**
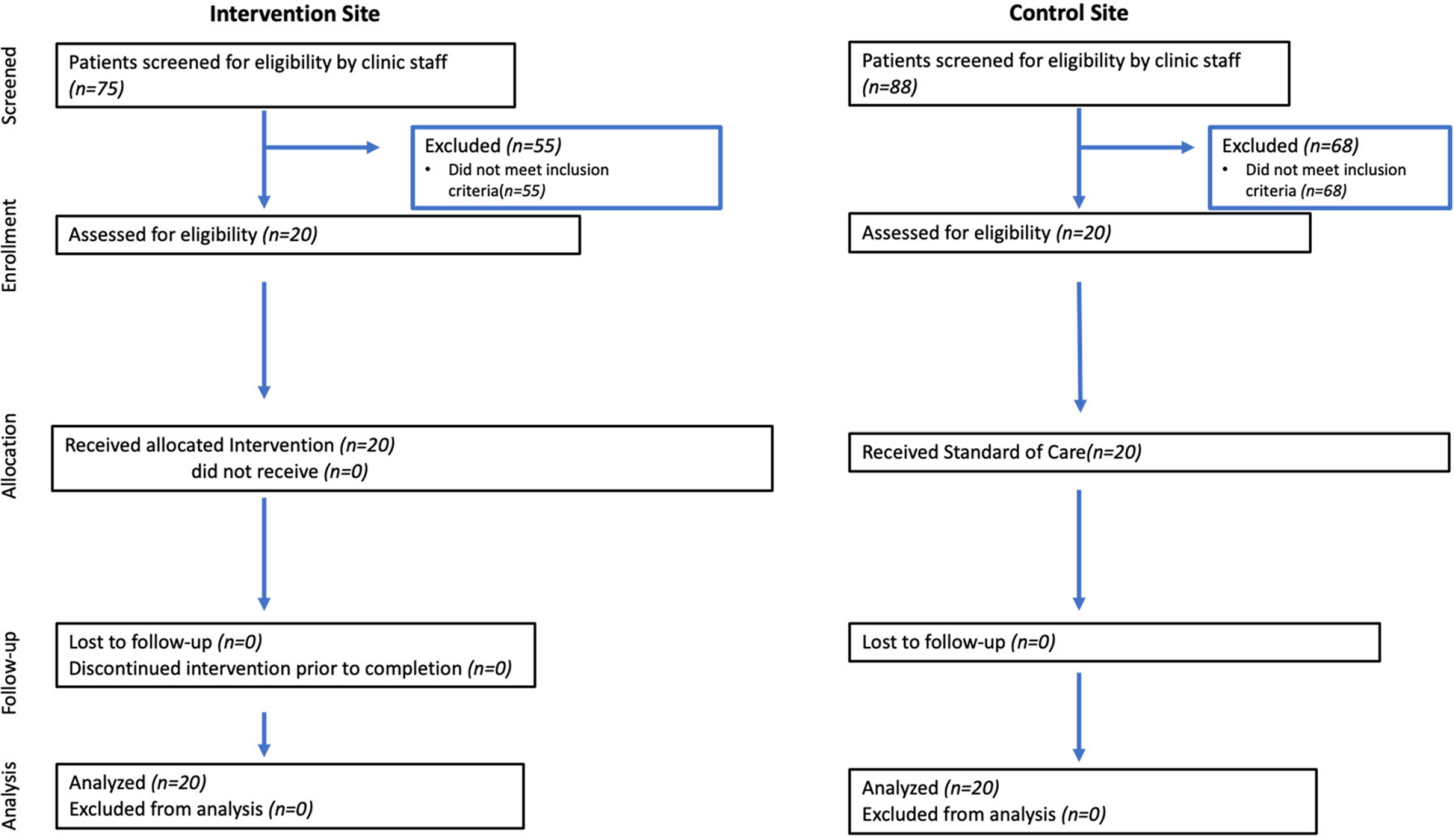
Consort pilot and feasibility flow diagram modified for Supporting Healthy Mothers

We analyzed 20 exit interviews, using the framework approach beginning with open coding where we applied short descriptive phrases or labels to women’s experiences or feedback about the intervention [42]. From the open coding, we then created an analytical framework—a set of codes (based on the labels applied during open coding) organized into categories. We then developed descriptive written summaries of the coded excerpts including outliers (comments or experiences pertaining to one/few participants). Finally, we categorized these summaries according to the following questions: 1) How was the unconditional cash transfer used? 2) What happened when financial insecurity was reduced for women? 3) What practical skills and knowledge were gained from the infant feeding support sessions that women could apply? 4) What was the perceived impact of the infant feeding support sessions? 5) Was the intervention acceptable to women (Fig. 1)?

## Results

### Participant characteristics

The mean age of participants was approximately 28 years at enrollment. Most had completed primary level education (60%), and 55% lived in informal housing (i.e., not regulated by the state in terms of building codes or safety regulations and/or no legally identified owner or address). The majority of our participants were married and living with their partners with 25 and 35% of participants’ partners having additional wives/partners in the intervention and control groups respectively (see Table 1).

**Table 1:** Baseline characteristics

	Intervention Group (*n* = 20)	Control Group (*n* = 20)
Mean age (years)	27.95	28.50
Level of education		
None	1 (5%)	0
Some primary	7 (35%)	7 (35%)
Primary graduate	2 (10%)	4 (20%)
Some secondary	2 (10%)	5 (25%)
Secondary graduate	5 (25%)	3 (15%)
Some college	3 (15%)	1 (5%)
Type of Housing		
Informal housing	11 (55%)	11 (55%)
Town apartment	3 (15%)	4 (20%)
Rural home	2 (!0%)	3 (15%)
Town house	0	2 (10%)
Other (rural rented house)	4 (20%)	0
Relationship with the father of the baby		
Married	12 (60%)	15 (75%)
Living together as married	4 (20%)	2 (10%)
Boyfriend who lives separately	2 (10%)	2 (10%)
Divorced		1 (5%)
No relationship, single	2 (10%)	
Currently living with father of baby?		
Yes	15 (75%)	16 (80%)
No	5 (25%)	4 (20%)
Baby’s father has other wives or lives with other women as if married?		
Yes	5 (25%)	7 (35%)
No	15 (75%)	10 (50%)
Unsure	0	3 (15%)
Primary Occupation		
Trading	5 (25%)	14 (70%)
Day labor	2 (10%)	4 (20%)
None	13 (65%)	2 (10%)
Average household income (KES)		
	10,800	13,935
Average needed per month to meet basic needs (KES)		
	15,035 (group mean)	17,350 KES (group mean)
Average travel time from home to the clinic		
	39 minutes	39.75 minutes
Was this a planned or unplanned pregnancy?		
Planned	4 (20%)	12 (60%)
Unplanned	16 (80%)	8 (40%)
First pregnancy?		
Yes	4 (20%)	3 (15%)
No	16 (80%_	17 (85%)
Number of years in HIV Care		
≤ 2 years	5 (25%)	7 (35%)
> 2 years	15 (75%)	13 (65%)

### Associations between the intervention and food security and depressive symptoms

**Fig. 2:**
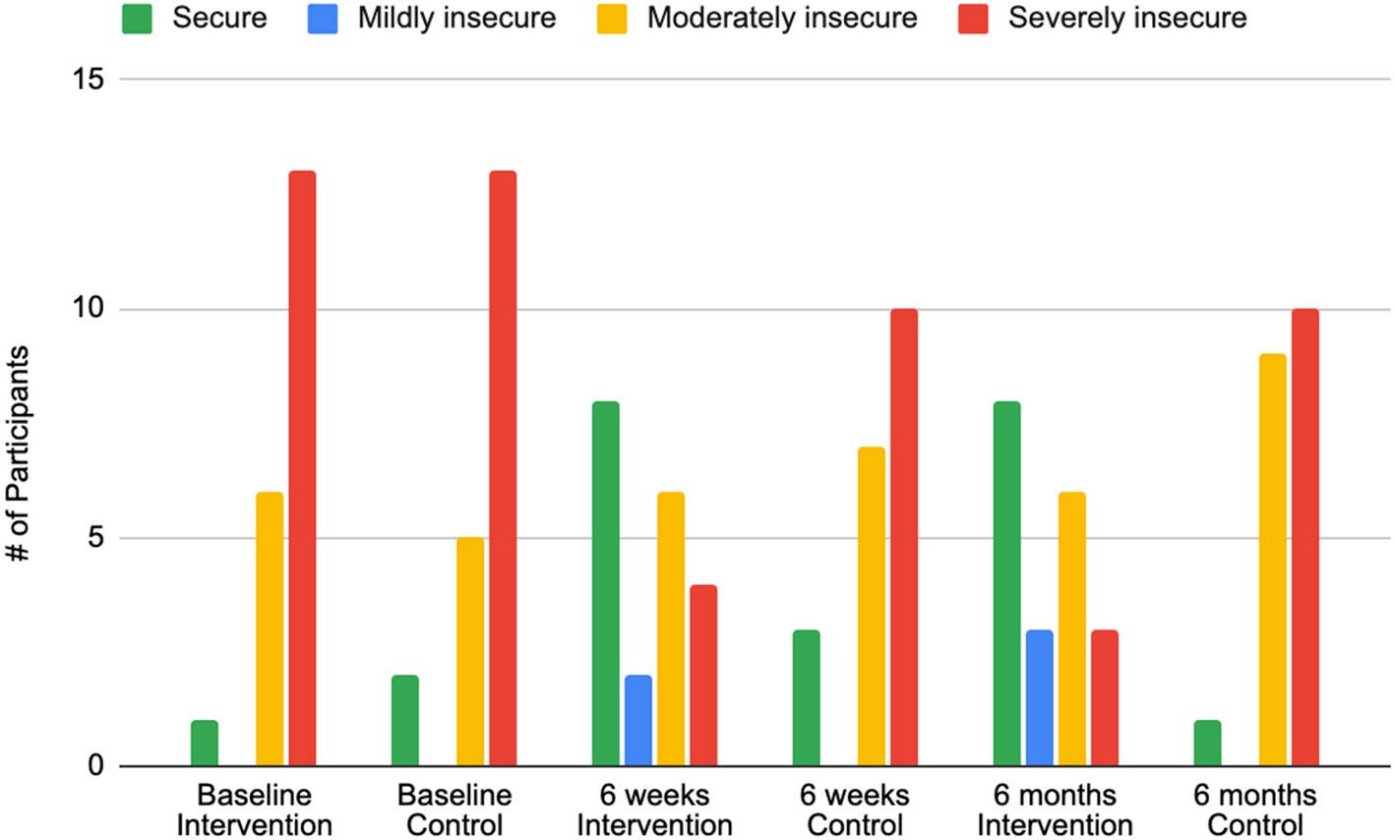
HFIAS scores by categories of food security

Food insecurity decreased during the study period with median scores of 11.5 and 2.0 for the intervention group compared to 12.0 and 9.0 for the control group at baseline and 6 months postpartum, respectively (see Table 2). There was a significantly greater reduction in food insecurity scores from baseline observed in the intervention group when compared to the control group at 6 weeks and 6 months postpartum (*p* = 0.0008 and *p* < 0.0001, respectively, Table 2). More than half of participants in each group were severely food insecure at baseline, but by 6 months postpartum, three (15%) women in the intervention group compared to 10 (50%) women in the control group were severely food insecure (see Table 2, see also Fig. 2 for categorized HFIAS scores across time).

**Table 2:** Measured scores and linear mixed model

Measured total scores	HFIAS	PHQ-9	PSS-10
Value	Median (IQR) [n]	Median (IQR) [n]	Mean (SD) [n]
**Intervention Group**			
Baseline	11.5 (9.0, 16.2) [20]	8.5 (6.0, 9.2) [20]	17.9 (6.4) [20]
6 weeks postpartum	1.0 (0.0, 7.0) [20]	3.0 (2.0, 6.2) [20]	12.2 (6.0) [20]
6 months postpartum	2.0 (0.0, 3.5) [20]	3.0 (1.0, 5.2) [20]	12.6 (6.3) [20]
**Control Group**			
Baseline	12.0 (7.0, 15.0) [20]	6.5 (5.0, 9.8) [20]	15.8 (8.4) [20]
6 weeks postpartum	9.5 (5.8, 12.5) [20]	3.0 (0.8, 5.0) [20]	12.2 (7.1) [20]
6 months postpartum	9.0 (6.0, 11.5) [20]	5.0 (1.0, 8.0) [20]	15.1 (6.7) [20]
**Linear mixed model**	**HFIAS**	**PHQ-9**	**PSS-10**
Baseline, all participants	10.74 (8.51, 12.86) [< 0.0001]	7.99 (6.21, 10.06) [< 0.0001]	15.85 (12.40, 19.70) [< 0.0001]
Intervention vs. Control	1.36 (−1.94, 4.61) [0.42]	0.06 (−2.45, 2.41) [0.94]	2.05 (−2.56, 6.54) [0.37]
All participants at 6 weeks postpartum (vs. baseline)	−1.93 (−4.43, 0.39) [0.11]	− 4.41 (− 6.48, − 2.56) [< 0.0001]	−3.54 (− 8.41, 1.38) [0.15]
All participants at 6 months postpartum (vs. baseline)	−1.50 (− 4.21, 1.20) [0.27]	−3.05 (− 5.75, − 0.39) [0.029]	−0.70 (− 5.36, 3.63) [0.77]
Change from baseline (Intervention vs. Control) at 6 weeks postpartum	−6.62 (− 10.14, − 3.17) [0.0008]	0.21 (−2.11, 2.68) [0.88]	− 2.21 (− 8.24, 3.75) [0.46]
Change from baseline (Intervention vs. Control) at 6 months postpartum	−8.05 (− 11.51, − 4.58) [< 0.0001]	− 1.40 (− 4.51, 1.84) [0.38]	−4.55 (− 10.00, 1.28) [0.12]

**Fig. 3:**
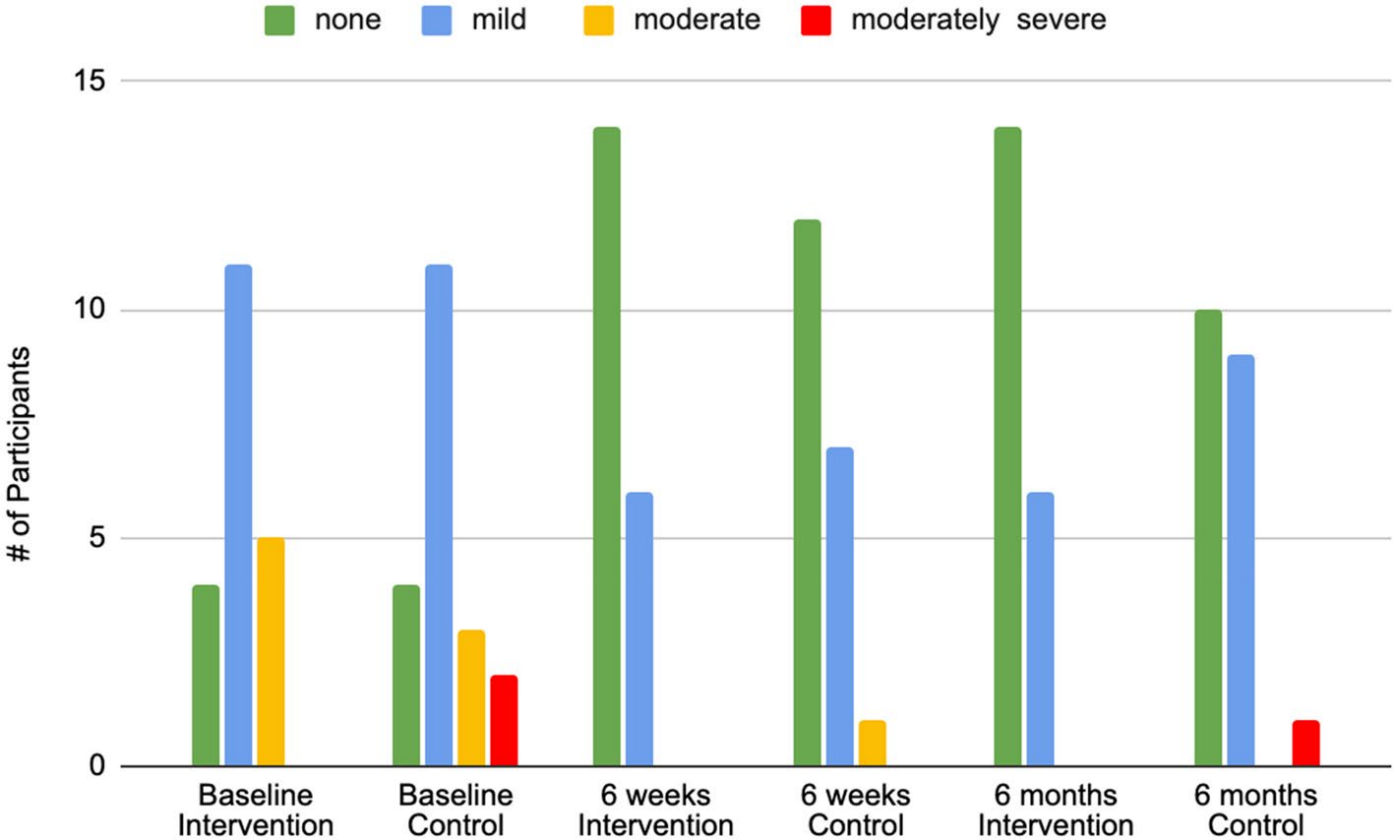
PHQ-9 scores by categories of depressive symptom severity

Median PHQ-9 scores for the intervention group decreased from 8.5 at baseline to 3.0 at 6 months postpartum, and in the control group median scores decreased from 6.5 to 5.0 between baseline and 6 months postpartum. The overall decrease in PHQ-9 scores for both groups was significant at 6 weeks and 6 months postpartum (*p* < 0.0001 and *p* = 0.029, respectively) but there was no significant difference in decrease between groups (see Table 2, see also Fig. 3 for categorized PHQ-9 scores across time).

Mean PSS scores decreased (indicating decreased stress) across the intervention period. For the intervention group, scores decrease from 17.9 at baseline to 12.6 at 6 months postpartum and for the control group scores decreased from 15.8 to 15.1 between baseline and 6 months (there was no statistically significant decrease or difference between groups, see Table 2).

### Breastfeeding behaviors

Most women in both groups planned to exclusively breastfeed at baseline, but around a quarter of women overall either did not believe they would produce enough breastmilk or were unsure. Follow-up surveys revealed that all women in the intervention group consistently reported feeding their babies “breastmilk only” while three (15%) women in the control group reported already giving their baby water at 2 weeks postpartum. In terms of perceived milk insufficiency, most women in both groups reported they were producing enough milk to satisfy their baby from 2 to 12 weeks postpartum. However, when asked if anything could help them to produce more breastmilk, most women in the control group answered, “yes, more food”, whereas nearly all women in the intervention group answered “no” (i.e., they were not lacking anything needed to support breastmilk production). At around 6 months postpartum, most women in the intervention group reported introducing complementary foods or had plans to begin doing so at 6 months postpartum or shortly thereafter. Meanwhile, less than half of the women in the control group reported introducing complementary feeding at 6 months postpartum with at least three women introducing earlier than recommended and nine women planning to wait until their baby was 7-months-old (see Table 3).

**Table 3:**
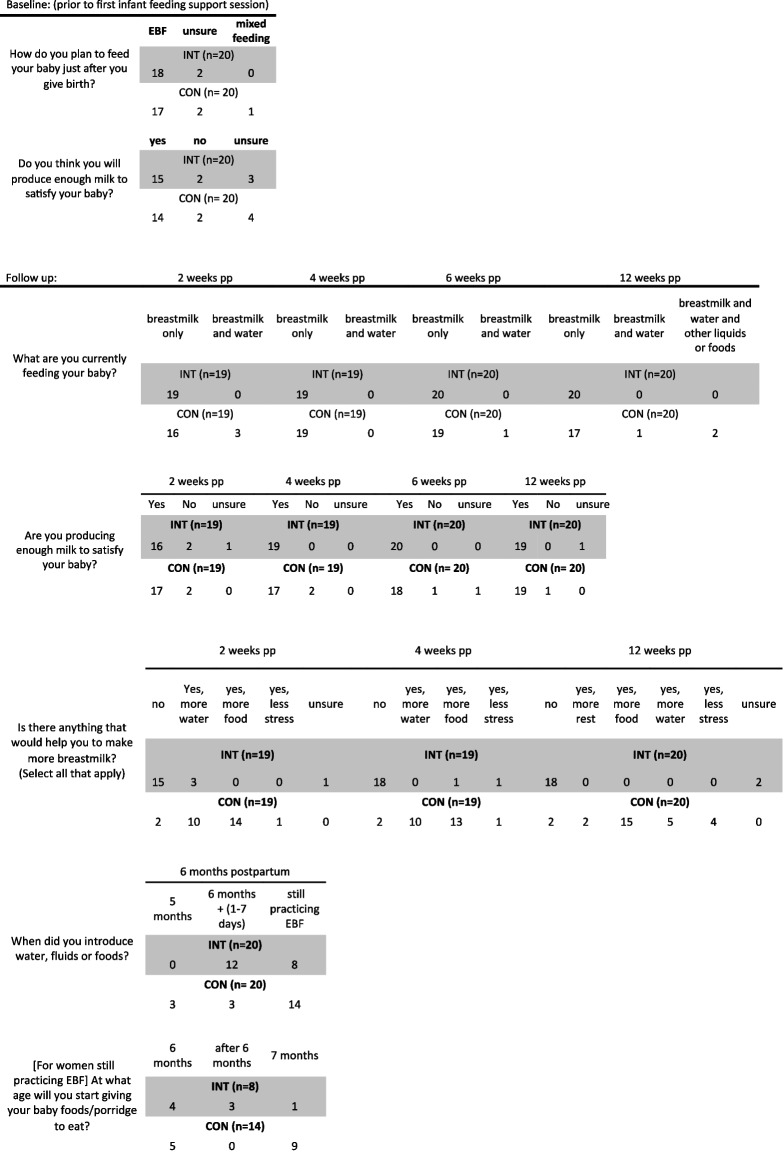
Baseline and follow-up questions about breastfeeding behaviors

### Exit interview findings

1. How was the unconditional cash transfer used?

Above all and without exception, women used the money to purchase food and water. In addition to these basic needs, most women also reported buying clothes, baby items, or other household items such as mattresses for sleeping or pots for cooking. Using the money for rent, school fees, medical bills, or for transportation to the clinic was also mentioned by most. More than a quarter of women used part of the funds to revive or start-up small businesses, and around the same number of women allocated money to support the needs of their close family members or church. Women also paid off debts and/or saved some of the money.

2. What happened when financial insecurity was reduced for women?

“It has changed my life” is how most participants described their experience with Supporting Healthy Mothers. Women explained, the money transfers had spared them from desperate circumstances such as needing to beg for money or food. Financial insecurity had been a primary stressor for women—straining relationships and leaving at least two women feeling so hopeless they had thoughts of ending their life.

*PID 18- It would have been bad [without the money]. I do not think this marriage would have survived…Because (sighs) you cannot be staying together, and the children are not going to school and you are just there and you know you also need food. The children also need food, they need clothes to put on, it would have been very tough, it would have been a tough life…(Sighs).*

According to participants, the funds provided instant relief from overwhelming worries, through increased food security, improved relationships and better options for caring for their infants and other children. Women explained that this relief brought them joy, happiness and hopefulness.

*PID 20: It relieved me from the stress I had in my heart. I can see the progress I have made, and it has helped me in so many ways.*

Several women, also mentioned using the funds to support their engagement in HIV care.

*PID 20: Yes. I am currently experiencing a lot of changes even when my VL [viral load] test is done, the levels are going down, my weight is good.*

The unconditional cash transfers also decreased women’s dependence on others and increased their ability to provide for their family, which together with the relief from stress led women to feel empowered, strong, motivated and courageous.

*PID 04- I just felt totally empowered. It has motivated me with my baby because you know when you lack, and you have a baby, sometimes you can be angry, you can react…overreact, but as of now, I was living a comfortable life.*

Naturally, women sought ways to sustain this financial security/ independence. Nearly all women were planning to engage in income generating activities or were already working by the time of the exit interview including eight of nine women who, at baseline, reported having no occupation or independent source of income even prior to pregnancy.

*PID 06- I used to work in a kiosk, I have a kiosk. I used to buy soap, detergent, sugar. It collapsed, and when I began receiving this money, I began saving bit by bit, I was not using it all. I would save little by little… I began stocking…Currently I have everything, I am selling charcoal, kerosene and the likes. I have developed with the help of this money, it has helped me by the way, yes.*

Most relationships between women and their partners improved during the study period. Over half of the women told their partners about the money either because they felt disclosure was unavoidable or because they wanted to tell. In contrast, many did not disclose or withheld details such as the amount being received or the number of payments because they feared their partners would stop providing or try to take control of the money. Among women who reported a lack of support or problems with partners during the intervention, all reported the problems were preexisting and/or unrelated to the money and no women reported being worse off in their relationship. Regardless of whether or not women told their partners about the money, relationships improved for most in various ways. Some women reported feeling closer to their partners as they collaborated to decide about how to spend the money.

*PID 15- Yes, very much. We became close, we could tell stories, we could do stuff together and he could even laugh whenever we were talking.*

For others, having the money made them feel more respected or valued by their partners.

*PID 18- He does not speak rudely to me because he knows I am everything. If a child was in need of anything, he can send the child to ask me for it because he knows I have it.*

Women also reported improved infant feeding experiences related to the unconditional cash transfers. For example, several women noted that improved food security facilitated adequate breastmilk production.

*PID 11- The intervention? Giving us that money helped us. I would go and buy food with that money because I know the baby had to breastfeed. So, I had to eat the food so that I can have plenty of milk for the baby to breastfeed and get satisfied.*

Other women noted the money allowed them (stress free) time to stay home from work, breastfeed and care for their baby or that it allowed them to purchase adequate complementary foods.

*PID 02- Since you started funding me, I have not been outside my door, I have not been leaving. So, people are like, (mentions her own name) stays at home, how has she been paying her rent? How does she feed when she is just home? You see, the stress of rent and how I feed is gone.*

When asked how they felt about the money transfers ending, most women described feeling positive, prepared or grateful. That said, some believed a bit more time supported by the money would put them in a better position to sustain themselves as their baby would be older and/or their business more established and income more stable. Others wished the money transfers would be extended to sustain optimal infant care—continued breastfeeding, optimal complementary feeding and more time with their baby before starting back to work.

*PID 13-* [explaining why the money should continue for another year] *R: Okay, sometimes someone has not organized himself/herself, how I was eating the balanced diet will reduce and the way to feed the baby good food as recommended can also reduce. Sometimes it will reduce, and she will be unable to access the food well, and she will not be able to get sufficient milk, so I do not know.*

3. What practical skills and knowledge were gained from the infant feeding support sessions that women could apply?

The majority of women were able to accurately recall what they learned about breastfeeding technique, including many technical skills such as how to position their baby, how to achieve an effective latch, how and why to burp their baby, how to understand whether or not their baby was getting enough milk and the importance of feeding on demand and giving their attention to their baby while breastfeeding. Most women also recalled key knowledge including the primary factors that impact milk production and the concepts of foremilk and hindmilk. Understanding foremilk and hindmilk provided women with a rationale for breastfeeding duration and frequency and for alternating between breasts. In addition, women explained that watery foremilk was evidence that their babies did not need water in addition to breastmilk (a commonly held belief among women locally) [43, 44].

*PID 09- the more the baby breastfeeds, the more milk is produced…. It signals the brain to produce milk when there is none, and the more you breastfeed, the more milk is produced. I felt that was true, because when I was breastfeeding her, despite how hungry I felt, more milk would still be produced whenever I breastfeed her.*

Women also credited the lactation specialist with teaching them about the many benefits of breastfeeding and specifically exclusively breastfeeding for the first 6 months.

*PID 07 - I had thought that I would wean her at 2 months because considering that I was going to leave her with someone else while I go to work. When I received the information, I continued breastfeeding her and I did not wean her until 6 months.*

Finally, nearly all women gained important (and new) information about complementary feeding including the types of food to give, how to prepare baby’s food and the consistency and quantity of food their baby needs at each stage.

*PID 10 - Actually before the lessons, before lessons, I didn’t know how to feed the baby. After giving me that balanced diet, that chart, yes, I know what to give and what not to give*.

4. What was the perceived impact of the infant feeding support sessions?

Women reported that the information and support given in the sessions about breastfeeding and their baby’s progress as well as the opportunity to talk about their concerns reduced their worries and made them feel more happy, confident, informed and capable.

*PID 11- You see, I normally have stress and I do not talk about it but whenever I come here, I tell you about my issues, I pour them out to you, I leave you with that burden…when I leave here, I am okay, I do not even remember those things (Laughter). But when I keep them to myself, they eat me up, it becomes a problem, and even eating is a problem because the stress affects me everywhere.*

A major concern the lactation specialist addressed for women was worry about mother to child transmission of HIV.

*PID 13- All along I was worried that I would infect the baby with HIV, so the information gave me courage and made me know that…it removed my worries that I previously had. It removed the worries that I had and made me courageous, and I just breastfed my baby until he was 6-months-old.*

The information, support, reassurance and encouragement women received was also critical to helping women resist social/cultural pressures that impede optimal infant feeding and health, including “plastic teeth” removal (a practice that involves a traditional provider cutting an infant’s gums or actually extracting the infant’s primary tooth buds while claiming to have removed “plastic teeth” that are allegedly plaguing the infant) [45, 46].

*PID 12- No, since you had told me about it [referring to plastic teeth removal], I am now an expert on them, I am an expert, I can tell someone else not to take the baby to the metal place [to the traditional healer who “removes plastic teeth”].*

Seeing their infants’ weight at the beginning of each session was additional reassurance for women that their baby was getting enough breastmilk and many women noted that this increased their confidence and contentment with their baby’s progress.

*PID 04- According to me, it was about weight gain, it made me so happy. I was happy with it because every month I went, I could see him grow, change, and gain weight and I became confident, and I was also happy.*

For some participants, knowledge gained through the sessions led to improved complementary feeding practices, such as not forcefully feeding the baby.

*PID 18- The information I got from the lactation specialist helped me big time, because with the other children, the older ones, I would begin forcefully feeding them porridge once they were 6-months-old. The lactation specialist informed us that we should not forcefully feed the baby, so I do not forcefully feed my last baby, I did not force him.*

Notably, several women were experiencing challenges with complementary feeding (baby not taking food or spitting up porridge) at the time of the exit interview and wished for continued support.

5. Was the intervention acceptable to women?

All women reported that the intervention was good just the way it was delivered, reporting that it helped, it changed their life, that there was nothing negative and/or that they felt supported. All women thought the intervention should continue helping women and/ or be expanded to reach other women (who are suffering).

With regards to the infant feeding support sessions: Most women were happy with the length and timing of the sessions, though a few would have liked to meet with the lactation specialist immediately after birth and some women wished for the sessions to continue until they were done breastfeeding or until they had overcome the challenges of complementary feeding. Women liked that the sessions were private/ personalized, that the lactation specialist and research coordinator held their baby, that refreshments were given, that they were free to call with concerns and that much more than the standard information and support were provided (i.e., much more in comparison to the PMTCT clinic).

With regards to the money transfers: More than half said, the money was a major help but that they needed more to meet all the basic needs of their house, with most saying 15–20,000KES ($110-145USD) would have been enough. Just less than half said the money was sufficient or more than sufficient for all basic needs. Most were happy with payments being delivered monthly and some specifically noted they appreciated that the money was given: without conditions, on time, at the beginning of the month and/or via M-PESA. M-PESA proved particularly effective and secure. Even in the two cases when women lost access to their phones, they were able to recover their funds, since the money is delivered to a specific registered phone number, and not connected to a physical phone or sim card. In terms of being socially acceptable, most women did not tell anyone except for their partner about the money, and no one disclosed to more than one or two close friends/ relatives. Women did not discuss the money with others for a variety of reasons. Some felt being associated with such an intervention would reveal their HIV status, others thought people might think the money came from “evil” and some preferred not to tell simply because they believed others would ask them to share the money. Several family members who heard about the unconditional cash transfers were indeed suspicious about the source of the funds but eventually accepted or came to understand that the money came from a benign source. Overall, disclosing or not disclosing information about the money received was not problematic for women. In fact, we screened women at every visit, asking “Was there any negative thing you experienced as a result of receiving this money?” and all women at every visit responded, “no”.

## Discussion

Our objective was to assess the feasibility, acceptability and potential impact of a multilevel intervention aimed at addressing financial insecurity and suboptimal infant feeding through an unconditional cash transfer and personalized, professional infant feeding support. Women found the two intervention components highly acceptable and described an overall positive impact on wellbeing. The intervention was feasible to implement with 100 % retention and did not require any adjustments or changes to the protocol. Our findings show a statistically significant reduction in food insecurity scores for the intervention group as compared to baseline and the control group. During exit interviews, women confirmed they felt more financially secure and confident to exclusively breastfeed because of their newly acquired practical knowledge and skills related to infant feeding. To our knowledge, this is the first multi-level intervention to address financial insecurity and suboptimal infant feeding.

Dramatic improvements in HFIAS (food insecurity) scores were directly aligned with exit interview findings that described profound relief from food and financial stress. Prior interventions using cash transfers among perinatal WLWH have been conditional, using smaller payments and mainly centered on impacting engagement in perinatal and HIV related care. In these cases, cash transfers have positively impacted appointment attendance among perinatal women in sub-Saharan Africa [47, 48]. This intervention differs from previous cash transfer interventions in that we provided a larger and unconditional cash transfer meant to cover basic needs (10,000 KES/ $75USD per month compared to 550–685 KES/$4–6 USD per visit). We found that addressing this major upstream barrier (financial insecurity) without conditions positively impacted food security and maternal and infant wellbeing downstream.

Women also reported positive changes within their relationships. This was in stark contrast to previous research in this setting that indicated that many relationships were strained or broken down during the perinatal period when women leaned heavily on their support systems to make ends meet [18].

The wealth of knowledge and skills women gained throughout the five infant feeding support sessions is of particular significance given even one gap in knowledge or technique can derail a woman’s breastfeeding experience. Many barriers have been identified that prevent women from maintaining breastfeeding practice in this setting such as perceived milk insufficiency [44, 49], positioning and latch [50], cultural norms or social pressures [44, 51, 52] and returning to the workforce [23, 52, 53]. In their scoping review of 59 publications investigating women’s perceptions and experiences breastfeeding, Beggs et al., 2021 found women were motivated and assumed it would be easy to breastfeed and yet most experienced challenges they were unprepared to overcome [54]. In Kenya, Andare et al. [53], found similar results among WLWH showing strong knowledge and acceptance to exclusively breastfeed, but still incomplete success in practice. Setting up women for success by acknowledging breastfeeding challenges while having supportive programs in place to overcome them is clearly warranted. In this way, women in the intervention group gained skills to overcome challenges, felt more confident and able to resist social pressures to introduce foods and fluids before 6 months, and expressed satisfaction with their breastfeeding experience.

Women credited both the unconditional cash transfer and the infant feeding support sessions with making them happy and hopeful, with the majority noting marked relief from stress and worry about both finances and their infant’s nutrition and health. These exit interview findings and the positive trend we observed in PHQ-9 and PSS-10 scores are consistent with findings from several previous studies in this setting which found improvements in psychological wellbeing among cash transfer recipients [25, 55, 56]. Our findings also align with several studies that point to a positive correlation between mental health and breastfeeding [57–59].

### Public health implications

In effect, Supporting Healthy Mothers provided women with financial security similar to paid maternity leave. In low- and middle-income countries, longer durations of paid maternity leave have been associated with lower infant mortality [60], and in Kenya, 3 months of paid maternity leave is mandated by law [61]. Unfortunately, women working in the informal sector (this applies to many women and the vast majority of the participants in our study) are not eligible for this benefit. Supporting Healthy Mothers shows the potential benefits of financial support across the perinatal period, calling for further research to evaluate the costs associated with providing a financial safety net compared to the short- and long-term benefits to health and wellbeing.

Our findings also point to the need for improved infant feeding counseling and support. Currently, in this setting, inadequate and inaccurate counseling is a documented barrier to optimal infant feeding [52], and standard care generally offers no assessment of practices or practical solutions for overcoming challenges/barriers [62, 63]. Meanwhile, lactation specialists (also referred to as lactation consultants) are underutilized despite providing low-cost, low-risk, non-invasive, evidence-based, high-impact interventions [64–67]. A lactation specialist’s support with position and latch during the early postpartum period may facilitate the development of an effective breastfeeding practice during a time that is critical to the establishment of the maternal milk supply [68]. Trusted relationships between mothers and lactation specialists, such as were developed during Supporting Healthy Mothers, may also help mothers to prioritize sound, evidence-base advice over deeply ingrained, long standing cultural practices which are often less safe (e.g., “plastic teeth” removal or force feeding) [63]. Recent efforts to improve infant feeding support, such as the Baby Friendly Community Initiative launched by the Kenyan Ministry of Health in 2016, have also shown great potential [69, 70]. However, there is little evidence of the program’s sustainment in this setting since funding for the program’s development and roll-out ended (December 2017) [50]. In sum, the potential impact of high-quality infant feeding support underscores the need to bolster healthcare teams including through lactation specialists and community counterparts, who are vital to sustaining and expanding such important initiatives.

### Limitations

Given our multi-component intervention and study design, we are unable to assess the lactation support sessions and unconditional cash transfer separately. The two components may contribute uniquely to perinatal women’s experiences and future research may be needed to understand the extent to which desired outcomes are impacted by each component. We also acknowledge the possibility that the control group received some benefits beyond standard of care that could have positively impacted their perinatal experiences such as reimbursement for travel expenses, positive interactions with our research coordinators on site, and seeing their infants’ weight at each research encounter.

Our study was limited to pregnant and postpartum women living with HIV on ART in Kisumu County, Kenya, and therefore findings may not be generalizable to other populations. That said, the potential benefits of both intervention components would likely hold true for other perinatal women in low-resource settings. Future trials, should consider offering additional infant feeding support sessions for up to 6 months and beyond to support women during the transition from exclusive breastfeeding to complementary feeding. Extended financial support and information about budgeting or small business management might also ensure more women sustain an income post intervention.

Despite these limitations, we believe our mixed-methods findings indicate that the Supporting Healthy Mothers intervention has great potential for impact and is highly acceptable to the target population.

## Conclusions

The Supporting Healthy Mothers intervention consisting of unconditional cash transfers and personalized professional infant feeding support was feasible and acceptable. We observed improvements in women’s food insecurity scores, and women reported the intervention positively affected their mental health and infant feeding experiences. The Supporting Healthy Mothers pilot intervention has potential to positively impact women during a vulnerable transition and beyond by increasing financial security, knowledge and skills, and should be assessed in larger trials.

## Data Availability

The datasets from the current study are available from the corresponding author on reasonable request. The qualitative data is not available for distribution.

## Abbreviations

WLWH: Women living with HIV
PMTCT: Prevention of mother to child transmission of HIV
ART: Antiretroviral Therapy

## References

[1] The-Sustainable-Development-Goals-Report-2022. New York, NY: United Nations; 2022. Contract No.: e-ISBN: 978–92–1-001809-8.

[2] Programme UND. The SDGs in Action: What are the Sustainable Development Goals 2023: https://www.undp.org/sustainable-development-goals?gclid=CjwKCAjwscGjBhAXEiwAswQqNB9-rEpJFi18ckJljZtkVEZymTQ2z-qWRbxYPeCtVLm2MbnTHPsgtRoCMkwQAvD_BwE.

[3] UNAIDS Data 2022. Geneva Switzerland: Joint United Nations Program on HIV/AIDS; 2022. https://www.unaids.org/en/resources/documents/2023/2022_unaids_data.

[4] IN DANGER: UNAIDS Global AIDS Update 2022. Geneva: Joint United Nations Programme on HIV/ AIDS; 2022. Report No.: Licence: CC BY-NC-SA 3.0 IGO.

[5] Ivers LC, Cullen KA. Food insecurity: special considerations for women. Am J Clin Nutr. 2011; 10.3945/ajcn.111.012617. 10.3945/ajcn.111.012617 PMC3226027 22089447

[6] Laraia BA, Siega-Riz AM, Gundersen C. Household food insecurity is associated with self-reported pregravid weight status, gestational weight gain, and pregnancy complications. J Am Diet Assoc. 2010; 10.1016/j.jada.2010.02.014. 10.1016/j.jada.2010.02.014 PMC3018748 20430130

[7] Guideline: updates on HIV and infant feeding: the duration of breastfeeding, and support from health services to improve feeding practices among mothers living with HIV. Geneva: World Health Organization and United Nations Children’s Fund; 2016. https://www.who.int/publications/i/item/9789241549707. 27583316

[8] Evans C, Jones CE, Prendergast AJ. HIV-exposed, uninfected infants: new global challenges in the era of paediatric HIV elimination. Lancet Infect Dis. 2016; 10.1016/S1473-3099(16)00055-4. 10.1016/S1473-3099(16)00055-4 27049574

[9] Chalashika P, Essex C, Mellor D, Swift JA, Langley-Evans S. Birthweight, HIV exposure and infant feeding as predictors of malnutrition in Botswanan infants. J Hum Nutr Diet. 2017; 10.1111/jhn.12517. 10.1111/jhn.12517 28960594

[10] Global strategy for infant and Young child feeding. World Health Organization; 2013.

[11] Dunkley E, Ashaba S, Burns B, O'Neil K, Sanyu N, Akatukwasa C, et al. "I beg you...Breastfeed the baby, things changed": infant feeding experiences among Ugandan mothers living with HIV in the context of evolving guidelines to prevent postnatal transmission. BMC Public Health. 2018; 10.1186/s12889-018-5081-x. 10.1186/s12889-018-5081-x PMC5789624 29378548

[12] Duran MC, Bosire R, Beima-Sofie KM, Igonya EK, Aluisio AR, Gatuguta A, et al. Women's autonomy in infant feeding decision-making: a qualitative study in Nairobi, Kenya. Matern Child Health J. 2021; 10.1007/s10995-021-03119-1. 10.1007/s10995-021-03119-1 PMC8062297 33544286

[13] Ickes S, Sanders H, Lemein H, Kinyua J, Singa B, McAnally KH, et al. Influences of exclusive breastfeeding among low-wage, working mothers in Kenya: perspectives from managers, healthcare providers, daycare directors, mothers, and fathers (OR30-02-19). Curr Dev Nutr. 2019; 10.1093/cdn/nzz044.OR30-02-19.

[14] Nabwera HM, Jepkosgei J, Muraya KW, Hassan AS, Molyneux CS, Ali R, et al. What influences feeding decisions for HIV-exposed infants in rural Kenya? Int Breastfeed J. 2017; 10.1186/s13006-017-0125-x. 10.1186/s13006-017-0125-x PMC5508793 28717383

[15] Webb-Girard A, Cherobon A, Mbugua S, Kamau-Mbuthia E, Amin A, Sellen DW. Food insecurity is associated with attitudes towards exclusive breastfeeding among women in urban Kenya. Matern Child Nutr. 2012; 10.1111/j.1740-8709.2010.00272.x. 10.1111/j.1740-8709.2010.00272.x PMC6860665 20874844

[16] Operto E. Knowledge, attitudes, and practices regarding exclusive breastfeeding among HIV-positive mothers in Uganda: a qualitative study. Int J Health Plann Manag. 2020; 10.1002/hpm.2966. 10.1002/hpm.2966 31849114

[17] Kenya HIV Estimates Report. Kenya Ministry of Health National Aids Control Council 2018 October 2018. https://nacc.or.ke/wp-content/uploads/2018/11/HIV-estimates-report-Kenya-20182.pdf.

[18] Tuthill EL, Maltby AE, Odhiambo BC, Akama E, Pellowski JA, Cohen CR, et al. "I found out I was pregnant, and I started feeling stressed": a longitudinal qualitative perspective of mental health experiences among perinatal women living with HIV. AIDS Behav. 2021; 10.1007/s10461-021-03283-z. 10.1007/s10461-021-03283-z PMC8126180 33997940

[19] Meleis AI (2010). Transitions theory: middle range and situation specific theories in research and practice.

[20] Leon AC, Davis LL, Kraemer HC. The role and interpretation of pilot studies in clinical research. J Psychiatr Res. 2011; 10.1016/j.jpsychires.2010.10.008. 10.1016/j.jpsychires.2010.10.008 PMC3081994 21035130

[21] Whitehead AL, Julious SA, Cooper CL, Campbell MJ. Estimating the sample size for a pilot randomised trial to minimise the overall trial sample size for the external pilot and main trial for a continuous outcome variable. Stat Methods Med Res. 2016; 10.1177/0962280215588241. 10.1177/0962280215588241 PMC4876429 26092476

[22] Tuthill EL, Maltby AE, Odhiambo BC, Akama E, Dawson-Rose C, Weiser SD. Resilient mothering: an application of transitions theory from pregnancy to motherhood among women living with HIV in western Kenya. ANS Adv Nurs Sci. 2023; 10.1097/ANS.0000000000000478. 10.1097/ANS.0000000000000478 PMC10354209 36656116

[23] Tuthill EL, Maltby AE, Odhiambo BC, Akama E, Dawson-Rose C, Cohen CR, et al. Financial and food insecurity are primary challenges to breastfeeding for women living with HIV in Western Kenya: a longitudinal qualitative investigation. AIDS Behav. 2023; 10.1007/s10461-023-04046-8. 10.1007/s10461-023-04046-8 PMC10577374 37043052

[24] Informal sectors skills and occupations survey (ISSOS): basic report. Kenya Bureau of National Statistics 2020.

[25] Haushofer J, Shapiro J. The short-term impact of unconditional cash transfers to the poor: experimental evidence from Kenya. Q J Econ. 2016; 10.1093/qje/qjw025. 10.1093/qje/qjw025 PMC7575201 33087990

[26] Kagucia EW, Ochieng B, Were J, Hayford K, Obor D, O'Brien KL, et al. Impact of mobile phone delivered reminders and unconditional incentives on measles-containing vaccine timeliness and coverage: a randomised controlled trial in western Kenya. BMJ Glob Health. 2021; 10.1136/bmjgh-2020-003357. 10.1136/bmjgh-2020-003357 PMC7845730 33509838

[27] Maltby AE, Odhiambo BC, Nyaura M, Shikari R, Tuthill EL. Feasibility, acceptability and lessons learned from an infant feeding intervention trial among women living with HIV in western Kenya. BMC Public Health. 2023. 10.1186/s12889-023-16794-2. 10.1186/s12889-023-16794-2 PMC10557183 37798696

[28] Harris PA, Taylor R, Minor BL, Elliott V, Fernandez M, O'Neal L, et al. The REDCap consortium: building an international community of software platform partners. J Biomed Inform. 2019; 10.1016/j.jbi.2019.103208. 10.1016/j.jbi.2019.103208 PMC7254481 31078660

[29] Harris PA, Delacqua G, Taylor R, Pearson S, Fernandez M, Duda SN. The REDCap Mobile application: a data collection platform for research in regions or situations with internet scarcity. JAMIA Open. 2021; 10.1093/jamiaopen/ooab078. 10.1093/jamiaopen/ooab078 PMC8435658 34527889

[30] Harris PA, Taylor R, Thielke R, Payne J, Gonzalez N, Conde JG. Research electronic data capture (REDCap)--a metadata-driven methodology and workflow process for providing translational research informatics support. J Biomed Inform. 2009; 10.1016/j.jbi.2008.08.010. 10.1016/j.jbi.2008.08.010 PMC2700030 18929686

[31] Obeid JS, McGraw CA, Minor BL, Conde JG, Pawluk R, Lin M, et al. Procurement of shared data instruments for research electronic data capture (REDCap). J Biomed Inform. 2013; 10.1016/j.jbi.2012.10.006. 10.1016/j.jbi.2012.10.006 PMC3600393 23149159

[32] Mwangi V, Owuor S, Kiteme B, Giger M, Jacobi J, Kirui O. Linking household food security and food value chains in north west Mt. Kenya. Sustainability. 2020; 10.3390/su12124999.

[33] Kimani-Murage EW, Schofield L, Wekesah F, Mohamed S, Mberu B, Ettarh R, et al. Vulnerability to food insecurity in urban slums: experiences from Nairobi, Kenya. J Urban Health. 2014; 10.1007/s11524-014-9894-3. 10.1007/s11524-014-9894-3 PMC4242851 25172616

[34] USAID (2007). Household food insecurity access scale (HFIAS) for measurement of food access: Indicator guide.

[35] Kroenke K, Spitzer R, Williams J (2001). The PHQ-9: validity of a brief depression severity measure. J Gen Intern Med.

[36] Musana JW, Cohen CR, Kuppermann M, Gerona R, Wanyoro A, Aguilar D, et al. Association of differential symptoms of stress to hair cortisol and cortisone concentrations among pregnant women in Kenya. Stress. 2020; 10.1080/10253890.2019.1696305. 10.1080/10253890.2019.1696305 31747807

[37] Cohen S, Kamarck T, Mermelsstein R. A global measure of perceived stress. J Health Soc Behav. 1983; 10.2307/2136404. 6668417

[38] Bates D, Mächler M, Bolker B, Walker S. Fitting linear mixed-effects models Usinglme4. J Stat Softw. 2015; 10.18637/jss.v067.i01.

[39] R Core Team (2021). R: a language and environment for statistical computing Vienna.

[40] Canty A, Ripley B. boot: Bootstrap R (S-Plus). Functions 2021.

[41] Davison AC, Hinkley DV (1997). Bootstrap methods and their application.

[42] Gale NK, Heath G, Cameron E, Rashid S, Redwood S. Using the framework method for the analysis of qualitative data in multi-disciplinary health researchUsing the framework method for the analysis of qualitative data in multi-disciplinary health research. BMC Med Res Methodol. 2013; http://www.biomedcentral.com/1471-2288/13/117. 10.1186/1471-2288-13-117 PMC3848812 24047204

[43] Mbagaya GM. Child feeding practices in a rural Western Kenya community. Afr J Prim Health Care Fam Med. 2009; 10.4102/phcfm.v1i1.15.

[44] Samburu BM, Kimiywe J, Young SL, Wekesah FM, Wanjohi MN, Muriuki P, et al. Realities and challenges of breastfeeding policy in the context of HIV: a qualitative study on community perspectives on facilitators and barriers related to breastfeeding among HIV positive mothers in Baringo County, Kenya. Int Breastfeed J. 2021; 10.1186/s13006-021-00385-1. 10.1186/s13006-021-00385-1 PMC8106855 33964950

[45] Mutai J, Muniu E, Sawe J, Hassanali J, Kibet P, Wanzala P. Socio-cuItural practices of deciduous canine tooth bud removal among Maasai children. Int Dent J. 2010; 10.1922/IDJ-2281Mutai05. 20476714

[46] Atim F, Nagaddya T, Nakaggwa F, N-Mboowa MG, Kirabira P, Okiria JC. Agony resulting from cultural practices of canine bud extraction among children under five years in selected slums of Makindye: a cross sectional study. BMC Oral Health. 2018; 10.1186/s12903-018-0599-y. 10.1186/s12903-018-0599-y PMC6081831 30086761

[47] Yotebieng M, Thirumurthy H, Moracco KE, Kawende B, Chalachala JL, Wenzi LK, et al. Conditional cash transfers and uptake of and retention in prevention of mother-to-child HIV transmission care: a randomised controlled trial. Lancet HIV. 2016; 10.1016/S2352-3018(15)00247-7. 10.1016/S2352-3018(15)00247-7 PMC5485848 26847230

[48] Vanhuyse F, Stirrup O, Odhiambo A, Palmer T, Dickin S, Skordis J, et al. Effectiveness of conditional cash transfers (Afya credits incentive) to retain women in the continuum of care during pregnancy, birth and the postnatal period in Kenya: a cluster-randomised trial. BMJ Open. 2022; 10.1136/bmjopen-2021-055921. 10.1136/bmjopen-2021-055921 PMC8739676 34992119

[49] Chikhungu LC, Bispo S, Rollins N, Siegfried N, Newell ML. HIV-free survival at 12–24 months in breastfed infants of HIV-infected women on antiretroviral treatment. Tropical Med Int Health. 2016; 10.1111/tmi.12710. 10.1111/tmi.12710 PMC5096069 27120500

[50] Kavle JA, Ahoya B, Kiige L, Mwando R, Olwenyi F, Straubinger S, et al. Baby-friendly community initiative-from national guidelines to implementation: a multisectoral platform for improving infant and young child feeding practices and integrated health services. Matern Child Nutr. 2019; 10.1111/mcn.12747. 10.1111/mcn.12747 PMC6635904 30748118

[51] Mohamed MJ, Ochola S, Owino VO. A qualitative exploration of the determinants of exclusive breastfeeding (EBF) practices in Wajir County, Kenya. Int Breastfeed J. 2020; 10.1186/s13006-020-00284-x. 10.1186/s13006-020-00284-x PMC7236358 32423487

[52] Nabakwe EC, Egesah O, Kiverenge-Ettyang GA. Maternal and health care workers' perspectives on exclusive breastfeeding in the context of maternal HIV infection, in Busia county, western Kenya: a mixed methods cross-sectional survey. Int Breastfeed J. 2022; 10.1186/s13006-022-00454-z. 10.1186/s13006-022-00454-z PMC8894571 35246178

[53] Andare N, Ochola S, Chege P. Determinants of infant feeding practices among mothers living with HIV attending prevention of mother to child transmission Clinic at Kiambu Level 4 hospital, Kenya: a cross-sectional study. Nutr J. 2019; 10.1186/s12937-019-0490-y. 10.1186/s12937-019-0490-y PMC6825715 31677638

[54] Beggs B, Koshy L, Neiterman E. Women's perceptions and experiences of breastfeeding: a scoping review of the literature. BMC Public Health. 2021; 10.1186/s12889-021-12216-3. 10.1186/s12889-021-12216-3 PMC8626903 34836514

[55] Junior JK, Arlene M, Ahn R. The perspectives of Young women in rural Western Kenya on unconditional cash transfers. Poverty Public Policy. 2016;

[56] Kilburn K, Thirumurthy H, Halpern CT, Pettifor A, Handa S. Effects of a large-scale unconditional cash transfer program on mental health outcomes of Young people in Kenya. J Adolesc Health. 2016; 10.1016/j.jadohealth.2015.09.023. 10.1016/j.jadohealth.2015.09.023 PMC4724529 26576822

[57] Pezley L, Cares K, Duffecy J, Koenig MD, Maki P, Odoms-Young A, et al. Efficacy of behavioral interventions to improve maternal mental health and breastfeeding outcomes: a systematic review. Int Breastfeed J. 2022; 10.1186/s13006-022-00501-9. 10.1186/s13006-022-00501-9 PMC9446548 36064573

[58] Mezzacappa ES, Katkin ES. Breast-feeding is associated with reduced perceived stress and negative mood in mothers. Health Psychol. 2002; 10.1037//0278-6133.21.2.187. 11950109

[59] Lara-Cinisomo S, D'Anna-Hernandez K, Fujimoto EM, Pedersen CA. Exploring associations between perinatal depression, anxiety, and urinary oxytocin levels in Latinas. Arch Womens Ment Health. 2019; 10.1007/s00737-018-0910-6. 10.1007/s00737-018-0910-6 PMC7141787 30191332

[60] Nandi A, Hajizadeh M, Harper S, Koski A, Strumpf EC, Heymann J. Increased duration of paid maternity leave lowers infant mortality in low- and middle-income countries: a quasi-experimental study. PLoS Med. 2016; 10.1371/journal.pmed.1001985. 10.1371/journal.pmed.1001985 PMC4811564 27022926

[61] Laws of Kenya: Employment Act Chapter 226. 2012. Available from: http://www.kenyalaw.org.

[62] Maryam ALM, Nadia SA, Rosemary K. Barriers to the practice of exclusive breastfeeding among HIV-positive mothers in sub-Saharan Africa: a scoping review of counselling, socioeconomic and cultural factors. J AIDS HIV Res. 2016; 10.5897/jahr2015.0353.

[63] Nyoni S, Sweet L, Clark J, Ward P. A realist review of infant feeding counselling to increase exclusive breastfeeding by HIV-positive women in sub Saharan-Africa: what works for whom and in what contexts. BMC Public Health. 2019; 10.1186/s12889-019-6949-0. 10.1186/s12889-019-6949-0 PMC6518720 31088541

[64] Teich AS, Barnett J, Bonuck K. Women's perceptions of breastfeeding barriers in early postpartum period: a qualitative analysis nested in two randomized controlled trials. Breastfeed Med. 2014; 10.1089/bfm.2013.0063. 10.1089/bfm.2013.0063 PMC3903167 24304033

[65] Chetwynd EM, Wasser HM, Poole C. Breastfeeding support interventions by international board certified lactation consultants: a systemic review and Meta-analysis. J Hum Lact. 2019; 10.1177/0890334419851482. 10.1177/0890334419851482 31206317

[66] Patel S, Patel S. The effectiveness of lactation consultants and lactation counselors on breastfeeding outcomes. J Hum Lact. 2016; 10.1177/0890334415618668. 10.1177/0890334415618668 26644419

[67] Haase B, Brennan E, Wagner CL. Effectiveness of the IBCLC: have we made an impact on the Care of Breastfeeding Families over the past decade? J Hum Lact. 2019; 10.1177/0890334419851805. 10.1177/0890334419851805 31206324

[68] Huang SK, Chih MH. Increased breastfeeding frequency enhances Milk production and infant weight gain: correlation with the basal maternal prolactin level. Breastfeed Med. 2020; 10.1089/bfm.2020.0024. 10.1089/bfm.2020.0024 32799538

[69] Kimani-Murage EW, Kimiywe J, Mutoro AN, Wilunda C, Wekesah FM, Muriuki P, et al. Effectiveness of the baby-friendly community initiative on exclusive breastfeeding in Kenya. Matern Child Nutr. 2021; 10.1111/mcn.13142. 10.1111/mcn.13142 PMC8189218 33528102

[70] Maingi M, Kimiywe J, Iron-Segev S. Effectiveness of baby friendly community initiative (BFCI) on complementary feeding in Koibatek, Kenya: a randomized control study. BMC Public Health. 2018; 10.1186/s12889-018-5519-1. 10.1186/s12889-018-5519-1 PMC5941766 29739374

